# Differences in HIV-related knowledge, attitudes, and behaviors among men who have sex with men (MSM): comparison between HIV-positive and HIV-negative college students

**DOI:** 10.3389/fpubh.2025.1672161

**Published:** 2025-11-28

**Authors:** Zhengwei Jing, Wenhui Chang, Hongyuan Wang, Chao Zhou, Tao Cui, Shiyao Xu, Peng Liao, Fei Li, Therese Hesketh, Yan Ning, Zhifeng Wang

**Affiliations:** 1 School of Public Health, Hangzhou Medical College, Hangzhou, Zhejiang, China; 2 Shaanxi Provincial Center for Disease Control and Prevention, Xi’an, Shaanxi, China; 3 School of Public Health, Peking University, Beijing, China; 4 Chongqing Center for Disease Control and Prevention, Chongqing, China; 5 School of Public Health, Fudan University, Shanghai, China; 6 Institute for Global Health, University College London, London, United Kingdom; 7 Chinese Center for Health Education, Beijing, China; 8 Center for Health Policy and Technology Assessment, Peking University Health Science Center, Beijing, China

**Keywords:** HIV, college students, men who have sex with men, associated factors, China

## Abstract

**Objective:**

This study aimed to compare HIV-related knowledge, attitudes, sexual behaviors, and social networking between MSM college students with and without HIV infection, and to identify associated factors sustaining the high HIV prevalence in this population.

**Methods:**

A cross-sectional survey was conducted among 686 HIV-positive and HIV-negative MSM college students recruited from Guangdong, Chongqing, and Shaanxi provinces (January 2019–March 2021) via local Centers for Disease Control institutions and Non-Governmental Organizations. Data were collected through structured face-to-face interviews assessing sociodemographic, HIV knowledge sexual practices, and social behaviors. Comparative analyses utilized *t*-tests and Barnard/Fisher exact tests (significance: *p* < 0.05), reporting effect sizes.

**Results:**

HIV-positive students were significantly older (median: 23 vs. 22 years; *p* = 0.007, *δ* = 0.14) and more likely to engage in off-campus MSM networking (*V* = 0.20, *p* < 0.001). Overall HIV knowledge awareness was 75.4%. However, it was significantly deficient in recognizing the interconnected epidemic risk between sexually transmitted infections and HIV. HIV-negative students reported higher rates of sexual activity with peers (*V* = 0.28, *p* < 0.001), favorable attitudes toward one-night stands (*V* = 0.13, *p* = 0.019), and stronger protective practices including disclosing HIV status pre-intercourse (25.2% vs. 9.1%; *V* = 0.21, *p* < 0.001), consistent condom use with casual partners (87.6% vs. 68.0%; *V* = 0.24, *p* < 0.001), and with partners met on-line (60.9% vs. 39.9%; *V* = 0.21, *p* < 0.001). Only 50.6% of online partnerships involved condom consistency.

**Conclusion:**

Persisting high HIV incidence among Chinese MSM students suggests deficits in translating HIV knowledge into protective behaviors, notably in on-line partner-seeking contexts. Tailored interventions must address structural vulnerabilities through integrated campus health services and technology-enabled risk-reduction strategies.

## 1 Introduction

HIV/AIDS remains a major public health problem in China (1, 2). During the early years of the HIV/AIDS epidemic in China in the 1990s the major at- risk group was injecting drug users (IDU) (3). In recent years, sexual transmission has emerged as the major route for HIV infection (3). Although men who have sex with men (MSM) have been recognized as a key population in the Chinese government’s HIV prevention strategies since the 2000s, MSM are now the highest-risk group for HIV infection in China, accounting for over 50% of new HIV infections in major Chinese cities (4, 5). Sentinel surveillance found that the national HIV prevalence among MSM in China was 8.0% in 2015, 200-fold greater than the 0.04% in the general Chinese population (6, 7). A major cause for concern has been the recent increase in prevalence of HIV in the student the MSM population in China. Reported HIV prevalence among MSM students across China has been increasing: 3.0% in 2003–2006, 4.5% in 2007–2008, and 6.8% in 2009–2010 (8). Between 2010 and 2019, 23,307 cases were reported in students, with a mean age at diagnosis of 19.9 (SD 2.05) years (9). Among these cases, 97.1% (22,640) were male, of whom 82.4% acquired the infection via homosexual contact. Most of these cases were identified at voluntary counseling and testing (VCT) clinics (9).

AIDS prevention interventions targeting university students in China operate within a three-tiered prevention framework. Primary prevention focuses on infection risk reduction through promoting condom use, pre−/post-exposure prophylaxis (PrEP/PEP), and behavioral interventions. Since 2015, the “University AIDS Prevention Fund” has supported over 1,100 student clubs across 730+ universities for peer-led prevention education; nationwide pilot programs further expanded PrEP/PEP implementation post-2019 to enhance epidemic control (10). Secondary prevention emphasizes early detection via routine testing, exemplified by a 2016–2019 initiative deploying urine-based self-testing kiosks in 73 universities across 11 provinces. Analysis of returned samples revealed a 2.3% HIV positivity rate (11). Tertiary prevention mitigates disease progression through sustained antiretroviral therapy (ART) and social support systems, including hospital-led follow-up care to minimize comorbidities.

The effectiveness of these interventions critically depends on target populations’ knowledge base and attitudinal factors. At the primary prevention level, inadequate risk perception undermines tool adoption: PrEP/PEP utilization remains constrained by low awareness and acceptance, particularly among key groups like MSM (12). Conversely, enhancing risk awareness via tools such as mobile-based risk predictors demonstrably reduced high-risk behaviors (13). For secondary prevention, testing barriers stem from privacy concerns and stigma-related fears; however, integrating self-testing with social media outreach effectively overcame these cognitive obstacles, significantly boosting testing coverage (14). Regarding tertiary prevention, ART adherence hinges on dual cognitive-attitudinal dimensions: insufficient scientific literacy erodes treatment confidence, while negative psychosocial factors require targeted psychological support. Optimized approaches integrating educational reinforcement and social mobilization systematically address implementation bottlenecks across all tiers (12, 14).

The notable vulnerability of university students, particularly young MSM, necessitates precisely targeted HIV prevention strategies. Consequently, investigating Knowledge, Attitudes, and Behaviors (KAB) specifically concerning this population within the distinct university environment is critically important. This foundational understanding is essential for designing interventions aimed at enhancing their HIV risk awareness and promoting effective prevention and control practices on campuses (15, 16). Previous research indicates significant discrepancies between risk-taking behaviors and HIV-related knowledge among MSM students in China. Nationwide meta-analyses demonstrate a high prevalence of unprotected anal intercourse (65.2%) and multiple sexual partnerships (58.2%) within this population (17). A pronounced dissonance between KAB is particularly evident. HIV/AIDS awareness reached 90.8% among college MSM students in Wuhan, while high-risk sexual behaviors remained prevalent (18). Similarly, PrEP awareness (88.7%) sharply contrasts with its actual utilization rate of 13.8% (19). Critically, significant context-dependent risk patterns were observed. Evidence from Zhejiang indicated that 89.2% of MSM students utilized online platforms for casual partner seeking, with condom usage rates significantly lower than among men who have sex with only women, and engagement in commercial sex was substantially higher (20).

Current research confronts significant limitations, chiefly characterized by geographically restricted sampling with predominant reliance on single-province datasets (17, 18, 20–22), in addition to a deficit in systematic comparisons of KAB parameters between HIV-positive and HIV-negative students (23). To bridge these research gaps, this investigation implements a multi-province sampling strategy (Guangdong, Chongqing, Shaanxi) incorporating HIV serostatus stratification controls. We conducted systematic comparisons of KAB profiles between HIV-positive and HIV-negative individuals within Chinese MSM student populations across these regions. As the first study performing serostatus-based KAB analyses at this geographic scale within this demographic, our work establishes a critical evidence foundation for developing regionally tailored precision interventions.

Consequently, comprehensive delineation of KAB patterns among young MSM is essential for strategies enhancing HIV-risk knowledge and attitudes and advance evidence-based prevention protocols in university settings. This study therefore aims to compare HIV related knowledge, attitudes and behaviors manifestations between seropositive and seronegative MSM students, to inform optimization of campus-specific HIV control initiatives.

## 2 Methods

### 2.1 Study design and participants

**Figure 1:**
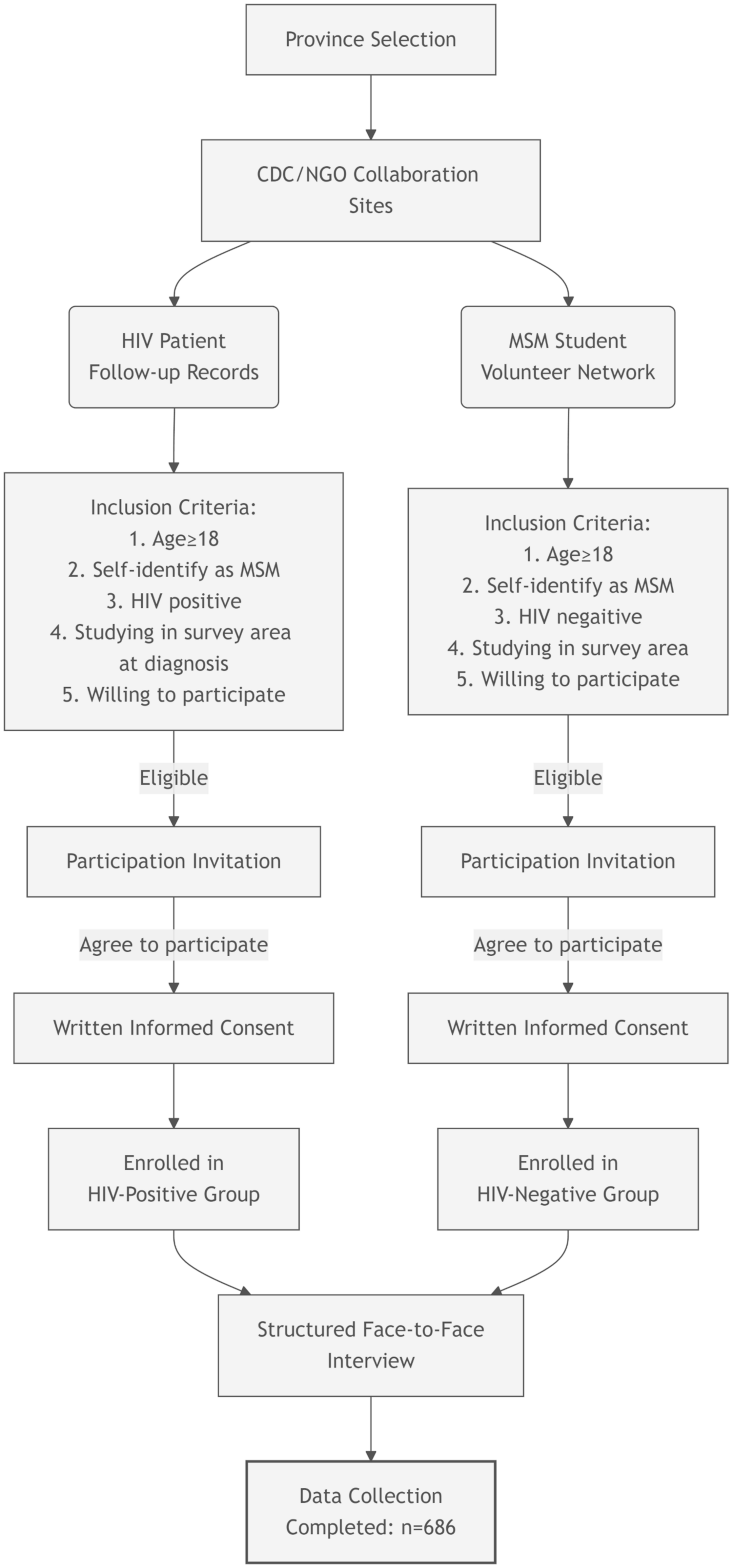
Recruitment flowchart.

The study design was a cross-sectional survey comprising a face-to-face interview. From January 19, 2019, to March 31, 2021, with the cooperation of the local Centers for Disease Control and Prevention and non-governmental organizations, MSM students were recruited from colleges and universities in Guangdong, Chongqing, and Shaanxi. According to provincial CDC data, some provinces in China have reported higher HIV infection rates among college students. Site selection criteria were determined based on expert consultation and approval from the HIV Surveillance Division at the Chinese Center for Disease Control and Prevention (China CDC). Participants’ HIV infection status was provided by the local CDC. To protect participant privacy, individuals were not required to disclose their university affiliation. The inclusion criteria were as follows: aged 18 years or over, self-identifying as MSM, and as HIV positive or negative, studying in the survey areas, and willingness to participate. The recruitment flowchart is shown in Figure 1.

### 2.2 Measurement tool

The questionnaire was developed by the research team drawing on existing research literature from China and other countries (24–36). The questionnaire underwent pilot testing, and subsequent adjustments were primarily focused on its structure and item content to enhance user-friendliness. The questionnaire comprised five sections: (1) Socio-demographic characteristics; (2) HIV-related knowledge was assessed using an 11-item instrument adapted primarily from the 2016 HIV Knowledge Questionnaire developed by China CDC. This adaptation incorporated all original items, while contextual modifications were informed by versions tailored for university students and MSM. All items employed a yes/no response format. Consistent with the China CDC’s criteria for HIV knowledge awareness, adequate knowledge was defined as ≥9 correct responses. The HIV-related Knowledge Scale demonstrated acceptable internal consistency, with a Kuder–Richardson Formula 20 (KR-20) coefficient of 0.784; (3) Health-seeking behaviors including HIV testing and treatment engagement. The health-seeking behavior subsection consisted of 12 items assessing HIV/AIDS-related testing, diagnosis, and treatment practices. Item formats included: five single-choice, four multiple-choice, and three open-ended items; (4) The sexual behaviors subsection included 37 items structured into five thematic domains: sexually transmitted infections (STIs) management, female partnerships, male partnerships, casual encounters, and transactional sex. Item formats featured 18 single-choice, 11 multiple-choice, and 9 open-ended items; and (5) Social behaviors were evaluated through 23 items across three venues: gay bars (8 items), bathhouses (7 items), and online platforms (8 items). Formats included nine single-choice, six multiple-choice, and eight open-ended items. The final three sections incorporated and adapted existing items where applicable, were piloted by the authors, and refined to create the final version.

### 2.3 Data collection procedure

Face-to-face interviews were conducted at collaborating non-governmental organization (NGO) offices providing tailored services for MSM, and government-designated hospitals with dedicated HIV counseling clinics. Interviewers underwent standardized training to implement neutral probing techniques and maintain non-judgmental responses during sensitive disclosures. Each interview session comprised two trained interviewers engaging exclusively with a single participant. To ensure data integrity and participant comfort, a hybrid survey methodology was employed: standardized HIV knowledge assessments were self-administered electronically by participants, while sociodemographic, behavioral, and attitude-related items were verbally administered and digitally recorded by one designated interviewer. Written informed consent was obtained from all participants in accordance with protocols approved by the institutional review board (IRB), which granted a waiver for documentary consent signatures to enhance privacy protection. Data completeness for all variables is detailed in Supplementary Table 2.

### 2.4 Statistical analysis

Questionnaire data were independently double entered using EpiData 3.0 to minimize keystroke errors, with all analyses conducted in R software. Socio-demographic characteristics are presented as frequencies (%) for categorical variables and mean ± standard deviation or median (interquartile range) for continuous variables based on Shapiro–Wilk normality tests. Intergroup comparisons employed independent samples *t*-tests (for normally distributed variables) or Mann–Whitney *U* tests (for non-normally distributed/heteroscedastic variables) for continuous data, and Barnard test (for 2 × 2 contingency tables) and Fisher’s exact test (for contingency tables larger than 2 × 2) for categorical variables; Effect sizes were reported as Cohen’s *d* or Cliff’s Delta for continuous variables, and Cramer’s *V* for categorical variables. Multiple comparisons were adjusted using the Benjamini–Hochberg method applied simultaneously to all intergroup test *p*-values. Statistical significance was defined as *p* < 0.05 (two-tailed).

### 2.5 Ethical approval and consent to participate

This study was approved by the Biomedical Ethics Committee of Peking University (Approval No: IRB00001052-18085), and informed consent was obtained from each participant before the survey was administered. Anonymity and confidentiality were guaranteed.

## 3 Results

Table 1 highlights statistically significant (*p* < 0.05) and clinically relevant contrasts, with complete datasets provided in Supplementary Tables 3–9. Specific characteristics are detailed in subsequent sections.

**Table 1:** Key sociodemographic, attitudinal, and behavior characteristics compared by HIV status among Chinese MSM College Students (Statistically significant differences only).

Variable	Overall	HIV-negative	HIV-positive	*p*-value	d/V^*^
	*N* = 686	*N* = 348	*N* = 338		
Age (median [IQR])	22.00 [21.00, 24.00]	22.00 [20.00, 24.00]	23.00 [21.00, 25.00]	0.007	0.14
How do you mainly meet friends in the MSM community? (%)					
MSM on-campus	237 (34.5)	153 (44.0)	84 (24.9)	<0.001	0.20
MSM off-campus	151 (22.0)	62(17.8)	89 (26.3)		
Both are the same	298 (43.4)	133 (38.2)	165 (48.8)		
Did you know the other person’s infection status before having sex? (%)					
Y	88 (17.0)	64 (25.2)	24 (9.1)	<0.001	0.21
N	348 (67.3)	145 (57.1)	203 (77.2)		
Partially known	81 (15.7)	45 (17.7)	36 (13.7)		
Do you want to know the other person’s infection status before having sex? (%)					
Y	491 (71.6)	282 (81.0)	209 (61.8)	<0.001	0.21
Do you protect yourself when having sex with a casual sex partner? (%)					
Y	535 (78.0)	305 (87.6)	230 (68.0)	<0.001	0.24
N	33 (4.8)	12 (3.4)	21 (6.2)		
Sometimes	118 (17.2)	31 (8.9)	87 (25.7)		
Attitude toward one-night stands (%)					
Approval	132 (19.2)	84(24.1)	48 (14.2)	0.019	0.13
Neutrality	353 (51.5)	165 (47.4)	188 (55.6)		
Be opposed to	201 (29.3)	99 (28.4)	102 (30.2)		
Sex role (%)					
Insertive	119 (17.3)	93 (26.7)	26 (7.7)	<0.001	0.26
Versatile	362 (52.8)	173 (49.7)	189 (55.9)		
Receptive	205 (29.9)	82 (23.6)	123 (36.4)		
First time men have sex with men (%)					
Mutual consent	605 (88.2)	317 (91.1)	288 (85.2)	0.045	0.13
Non-consensual	22 (3.2)	9 (2.6)	13 (3.8)		
Inducement by the other	45 (6.6)	13 (3.7)	32 (9.5)		
Others	14 (2.0)	9 (2.6)	5 (1.5)		
Number of male sex partners who are students (median [IQR])	2.00 [1.00, 4.00]	3.00 [1.00, 5.00]	2.00 [1.00, 4.00]	<0.001	−0.18
Number of male sexual partners in the past 6 months (%)					
0	232 (33.8)	74 (21.3)	158 (46.7)	<0.001	0.28
1	273 (39.8)	157 (45.1)	116 (34.3)		
2–5	160 (23.3)	102 (29.3)	58 (17.2)		
>5	21 (3.1)	15 (4.3)	6 (1.8)		
Have a regular sexual partner in the past 6 months (%)					
Y	354 (51.6)	212 (60.9)	142 (42.0)	<0.001	0.19
Having a casual sexual partner in the past 6 months (%)					
Y	199 (29.0)	129 (37.1)	70 (20.7)	<0.001	0.18
Do you want to have a long-term male sexual partner? (%)					
Y	599 (87.3)	315 (90.5)	284 (84.0)	0.047	0.10
Will you go to a gay bar in the future? (%)					
Y	193 (28.1)	127 (36.5)	66 (19.5)	<0.001	0.22
N	312 (45.5)	125 (35.9)	187 (55.3)		
Uncertain	181 (26.4)	96 (27.6)	85 (25.1)		
Condom use when having sex with men using software or forums (%)					
Keep using	305 (50.6)	187 (60.9)	118 (39.9)	<0.001	0.21
Used partially	281 (46.6)	112 (36.5)	169 (57.1)		
Never use	17 (2.8)	8 (2.6)	9 (3.0)		
Will you visit gay social software or forums in the future? (%)					
Y	453 (66.0)	263 (75.6)	190 (56.2)	<0.001	0.21
N	98 (14.3)	30 (8.6)	68 (20.1)		
Uncertain	135 (19.7)	55 (15.8)	80 (23.7)		

*d, Cliff’s Delta effect size; V, Cramer’s V coefficient.

For continuous variables, Mann–Whitney U tests were used for non-normally distributed and/or heteroscedastic data. For categorical variables, Barnard tests were employed for 2 × 2 contingency tables, and Fisher’s exact tests for larger contingency tables. Effect sizes were reported as Cliff’s δ for continuous variables and Cramer’s V for categorical variables. Multiple comparisons were corrected using the Benjamini & Hochberg method. Statistical significance was defined as *p* < 0.05 (two-tailed). The selected cutoff points were |Cliff’s *δ*| ≥ 0.15 and Cramer’s V ≥ 0.10, indicating a small effect size according to conventional guidelines.

### 3.1 Socio-demographic characteristics

Completed data was obtained from 686 MSM college students; 348 (50.7%) were HIV-negative and 338 (49.3%) HIV-positive. Supplementary Table 3 shows the socio-demographic data by HIV status. Participants were from Guangdong (34.4%), Chongqing (33.5%), and Shaanxi (32.1%), with no significant differences in HIV positive prevalence across the samples in the three provinces (*p* = 0.573). The median age in the HIV-positive group was significantly higher [23.00 (21.00, 25.00) years] than the HIV-negative group [22.00 (20.00, 24.00) years, *p* = 0.007]. The overwhelming majority (86.2%) were undergraduate students with the remainder postgraduates (13.8%). 13.7% of participants reported rural residence prior to college.

### 3.2 Knowledge

Supplementary Table 4 summarizes HIV knowledge item-specific correct response rates among participants. The overall HIV/AIDS knowledge awareness rate was 75.4%, with no significant difference observed between HIV-positive (77.2%) and HIV-negative individuals (73.6%) (*p* = 0.476). Knowledge levels exhibited substantial variation across domains: The highest awareness was noted for condom efficacy (95.6%) and post-exposure testing recommendations (95.2%), while recognition of the interconnected epidemic risk between STIs and HIV scored lowest (59.2%).

### 3.3 Meeting sexual partners

Supplementary Table 5 reveals significant differences (*p* < 0.001) in meeting and socializing preferences among MSM by HIV serostatus. HIV-negative students were more inclined to make connections with on-campus homosexual peers (44.0% vs. 24.9%), whereas HIV-positive students showed preference for socializing with homosexuals off-campus (26.3% vs. 17.8%). Significant differences in protective behaviors were observed by HIV serostatus (*p* < 0.001 for all comparisons). Compared to HIV-positive peers, HIV-negative individuals were significantly more likely to: express a desire to know new partners’ infection status (81.0% vs. 61.8%), disclose asking about a partner’s HIV status prior to intercourse (25.2% vs. 9.1%), and report consistent self-protection during casual sexual encounters (87.6% vs. 68.0%).

### 3.4 Attitudes to high risk sexual behaviors

Supplementary Table 6 shows substantial proportions of MSM students held non-negative attitudes toward one-night stands and commercial sex. Regarding high-risk sexual behaviors, the acceptability of one-night stands was reported by 24.1% of HIV-negative individuals compared to 14.2% of HIV-positive individuals (*p* = 0.019).

### 3.5 Sexual behavior

**Figure 2:**
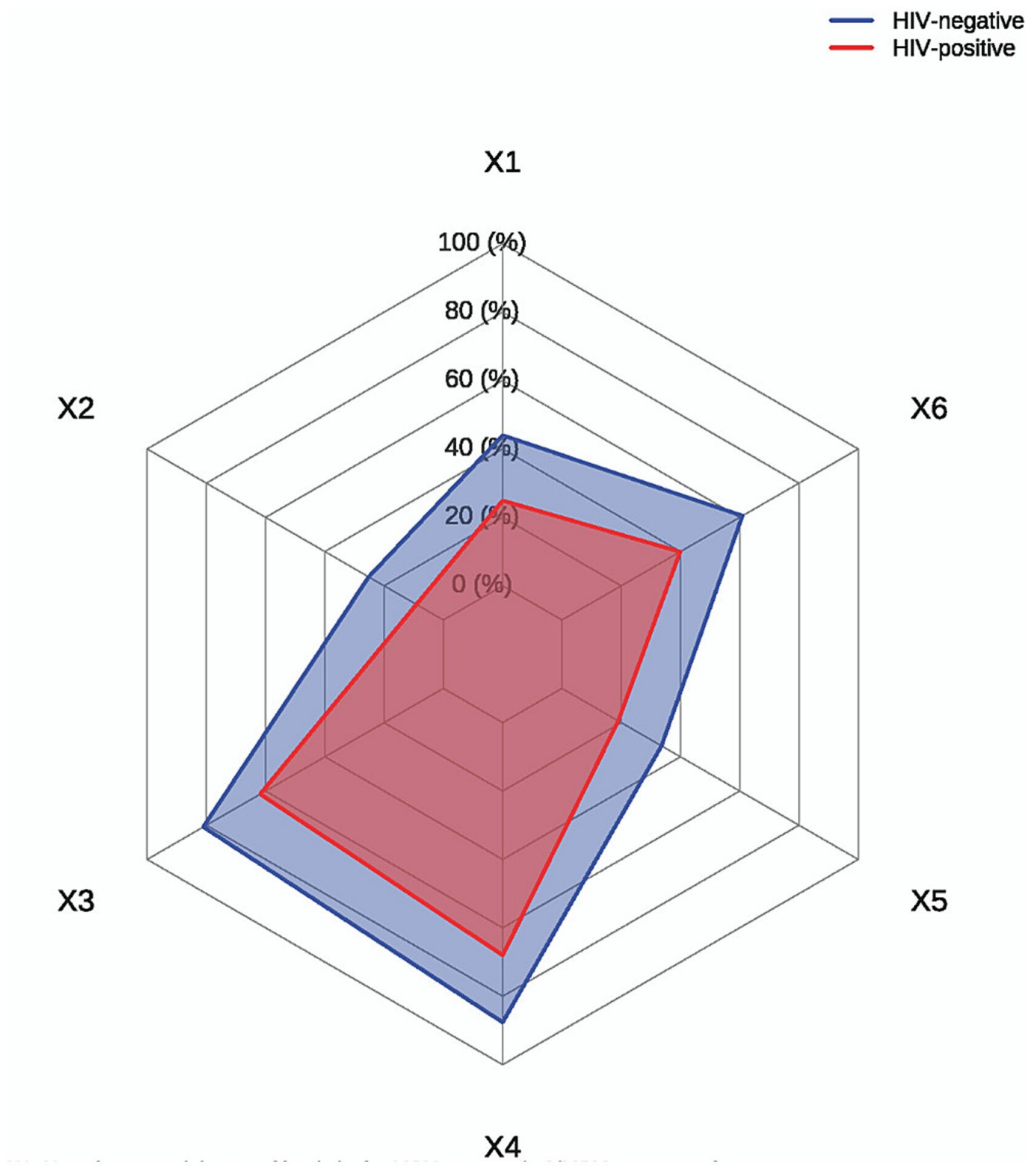
HIV-related behaviors and attitudes comparison. X1: How do you mainly meet friends in the MSM community?(MSM on-campus); X2: Did you know the other person’s infection status before having sex?(Y); X3: Do you want to know the other person’s infection status before having sex?(Y); X4: Do you protect yourself when having sex with a casual sex partner?(Y); X5: Number of male sexual partners in the past 6 months (>1); X6: Condom use when having sex with men using software or forums (Keep using).

Supplementary Table 7 presents self-reported sexual behavior profiles among participants. The median reported age at first sexual intercourse was 18.00 years, with 3.2% of initial sexual experiences reported as non-consensual. Only 66.2% of participants utilized condoms during their first sexual encounter. Significantly more HIV-negative students engaged in recent sexual activity, with higher proportions reporting both regular (60.9% vs. 42.0%; *p* < 0.001) and casual sexual partners (37.1% vs. 20.7%; *p* < 0.001) within the past 6 months relative to HIV-positive peers. Notably, the HIV-negative group had significantly higher numbers of student sexual partners compared to the HIV-positive group [3.00 (1.00, 5.00) vs. 2.00 (1.00, 4.00), *p* < 0.001], who were more active off-campus. Regarding condom utilization in the past 6 months, 68.2% of participants reported consistent condom use during all sexual acts. The two most frequently cited reasons for anal intercourse without condom protection, were “perceived partner HIV status” (10.2%) and “engagement in stable partnerships” (9.2%). Furthermore, 11.5% of participants had a history of STIs, with 96.2% having been successfully treated. Key behavioral differences are shown in Figure 2.

### 3.6 Social behavior related to sex for MSM

Supplementary Table 8 presents behavioral characteristics related to offline venues (e.g., gay bars and bathhouses) among participants. Of the totals 38.5% of HIV-negatives and 44% of HIV-positives reported having visited gay bars, with leisure and recreation cited as the primary purpose. Among bar attendees, 22 (7.8%) reported engaging in sexual activity within these venues, of whom 72.7% consistently used condoms. Separately, 72 participants (10.5%) had visited gay bathhouses, with 15 (20.8% of bathhouse visitors) reporting sexual encounters; within this subgroup, 10 individuals (66.7%) maintained consistent condom use.

Supplementary Table 9 delineates online behavioral patterns among participants. 96.9% of participants engaged with gay social media or forums. The three primary purposes for this engagement were leisure activities (54.1%), social networking (49.8%), and partner-seeking (36.5%). Notably, 90.7% of social media users reported sexual encounters with individuals met through these platforms, yet only 50.6% practiced consistent condom use. Moreover, HIV-positive individuals demonstrated significantly lower rates of consistent condom use during sex with partners sourced through digital platforms (39.9% vs. 60.9%, *p* < 0.001).

## 4 Discussion

This cross-sectional study investigated the association between HIV status and knowledge, attitudes and behaviors in a sample of 686 MSM college students in three Chinese provinces.

Overall HIV/AIDS knowledge awareness was moderate, indicating significant gaps. Awareness was particularly low regarding the association between STIs and increased HIV risk. Critically, the absence of a significant difference in HIV knowledge between HIV-positive and HIV-negative individuals suggests that the level of knowledge measured in this study was not associated with prevalent HIV infection in this cohort. This finding highlights a potential disconnect between HIV-related knowledge and protective behaviors (37, 38). This notable disconnect may originate from dimensions of the Health Belief Model (HBM) that were not comprehensively measured in the present study. Although the assessed knowledge framework encompassed critical HBM components including perceived susceptibility and perceived severity, which often constitute central foci in public health education campaigns (39). However, equally vital HBM constructs such as perceived barriers, cues to action, and self-efficacy warrant consideration. Specifically, persistent perceived barriers to protective behaviors were evident, including restricted access to prophylactics on campus, stigma hindering serostatus negotiation, and perceived reductions in sexual pleasure associated with condom use (40). Concurrently, the deficiency in cues to action undermined the impetus for knowledge translation. For instance, high-frequency use of online dating platforms was not complemented by integrated, HIV risk alert systems. Furthermore, self-efficacy development emerged as crucial: while awareness of risk-reduction strategies, such as condom utilization, was relatively high, observed deficiencies—particularly in negotiating protective behaviors with partners met on-line—revealed participants’ limited confidence and practical capacity to translate knowledge into protective actions within complex interpersonal contexts.

The findings reveal strong associations between HIV status and preferences for meeting partners and protective behaviors (41). HIV-negative students showed a significant preference for meeting partners on campus and reported significantly higher frequencies of behaviors generally associated with protection: an expressed desire to know partner’s status, disclosure of discussing status before sex, consistent self-protection during casual sex, and significantly higher levels of consistent condom use specifically with partners met online. In contrast, HIV-positive students were more likely to report off-campus socializing and partner seeking. While not implying causality, these associations suggest distinct patterns of sexual networking and protective practice related to their current HIV status. The notably low rates of discussing HIV status prior to intercourse in both groups highlight a persistent barrier to prevention (42).

Contrary to expectations that higher-risk attitudes might be associated with HIV infection, HIV-negative participants reported significantly higher acceptability of one-night stands, with significantly higher reports of recent sexual activity, both in terms of regular sexual partners and casual sexual partners within the past 6 months. Furthermore, the HIV-negative group reported significantly higher numbers of student sexual partners. A higher proportion of HIV-negative participants also reported recent casual sex. This complex picture indicates that the HIV-negative group appeared more sexually active within the campus environment but simultaneously reported better adoption of certain protective measures like consistent condom use with online partners. However, the association between higher partner numbers and negative status warrants careful interpretation; factors like more frequent testing behaviors in the HIV-negative group could be influential (43). Individuals who test negative for HIV routinely are more likely to be more confident in forming sexual partnerships, perhaps driven by a perception of lower personal risk or affiliation within social networks perceived as ‘safer’. Conversely, individuals diagnosed with HIV, particularly those undergoing treatment may exhibit reduced rates of new partnership formation. This reduction could stem from priorities related to managing their health condition, concerns about the physical impact of HIV, or anxieties associated with disclosing their serostatus to potential partners. Furthermore, the low consistent condom use during first sex across the cohort suggests vulnerability at the initiation of sexual activity, indicating a crucial intervention point.

The pervasive use of gay social media/forums among participants, primarily for leisure, networking, and partner seeking, underscores its centrality in the sexual lives of MSM students. The high rate of sexual encounters stemming from these platforms paired with a low rate of consistent condom use – significantly lower in HIV-positive users – highlights online spaces as a critical environment for potential HIV transmission and an essential avenue for prevention interventions within this demographic. Our finding supports that of a Chinese survey, showing that all HIV-positive MSM students who became infected through casual sex had sought sexual partners online (20). Behaviors in offline venues suggested that while a smaller proportion engaged in sex within these settings, condom use among those who did was relatively high. The primary purpose of visiting bars was reported as leisure, associating these venues more strongly with socialization than explicit partner-seeking for this sample.

### 4.1 Limitations

This study has several important limitations. First, the cross-sectional design inherently limits our findings to observed associations, precluding definitive conclusions regarding causality. We cannot determine the directionality of the relationships between identified factors and HIV serostatus. Second, observed characteristics such as higher sexual activity or partner numbers within the HIV-negative group may be confounded by factors like their significantly younger age distribution, or potentially earlier stage in their sexual life course (44, 45). Variables showing statistical significance in univariate comparisons were further subjected to age-adjusted analyses, with results presented in Supplementary Tables 10–12. Third, this study collected self-reported behavioral data via face-to-face interviews. As with all self-reported sensitive data, results are susceptible to recall and social desirability biases (46). While anonymity reduced bias, confidentiality prevented partner/clinic record verification. Results require cautious interpretation, especially for highly stigmatized behaviors. Fourth, although data were collected across three diverse Chinese provinces, the findings may not generalize to all MSM student populations in China or other cultural contexts. Fifth, our sampling strategy, which included HIV-negative MSM, with both regular and irregular clinic attendance, alongside HIV-positive individuals engaged with Centers for Disease Control programs, may overrepresent individuals with higher self-reported HIV knowledge and favorable perceptions, potentially further limiting the broader generalizability of these findings. Finally, the inability to document response rates due to privacy protocols further compounds potential participation bias, as we cannot assess whether non-respondents systematically differed in attitudes, behaviors, or risk profiles, potentially overrepresenting motivated or accessible participants and compromising estimates.

### 4.2 Implications for public health policy

Despite its methodological limitations, this study offers insights for refining HIV prevention strategies targeting college-based MSM in China. Critically, the finding that HIV knowledge was comparable across serostatus groups underscores a need for interventions that transcend knowledge dissemination. Effective programs must actively cultivate behavioral skills, bolster self-efficacy in prevention practices, and remove context-specific barriers, such as ensuring accessible prevention tools and reshaping normative attitudes surrounding HIV prevention within this population. In addition, near-ubiquitous online platform engagement needs to integrate technology-enabled risk-reduction strategies. Further, by amplifying the central role of institutions, the campus environment itself proves crucial. The concentration of partner-seeking among HIV-negative students on campus and heightened vulnerability linked to early-stage sexual experiences necessitate integrating enhanced MSM-affirmative, accessible, and confidential sexual health services and tailored peer-led education programs directly within university health frameworks (47, 48). Addressing the reluctance to disclose serostatus requires dedicated normalization initiatives that reduce stigma and enhance communication efficacy (47). Finally, we must recognize distinct differences in risk environments. Online venues are disproportionately associated with decreased condom use. Since most connections originate online, interventions should prioritize this domain with context-specific strategies.

## 5 Conclusion

This study reveals a complex pattern of associations linking HIV status among MSM college students to differences in age, meeting preferences, protective behaviors, sexual activity levels, and attitudes. The absence of a significant association with overall HIV knowledge reinforces the need for effective interventions in this population and must extend beyond information provision to actively address behavioral and socio-sexual factors that drive risk-taking, particularly online. Future longitudinal studies are needed to elucidate causal pathways and the impact of targeted interventions addressing the specific vulnerabilities identified. Tailored, culturally appropriate, and campus-integrated strategies that build skills, promote communication, normalize prevention, and leverage the digital landscape are vital for curbing HIV transmission in this vulnerable and important demographic.

## Data Availability

The datasets presented in this article are not readily available because of ethical restrictions. Requests to access the datasets should be directed to ZJ, jzw1993vivian@163.com.
