# Supplementary material for: Differences in HIV-related knowledge, attitudes, and behaviors among men who have sex with men (MSM): comparison between HIV-positive and HIV-negative college students
Source: Front Public Health. 2025 Nov 28;13:1672161. doi: 10.3389/fpubh.2025.1672161 (PMC12698562; doi:10.3389/fpubh.2025.1672161)
Supplement: Supplementary file 1 [file Supplementary_file_1.docx]

Supplementary Table 1. Key Term Definitions

| **Term** | **Definitions** |
| --- | --- |
| Off-campus networking | Participants are predominantly seeking non-student MSM individuals as sexual partners. |
| On-campus networking | Participants predominantly seek student MSM individuals as sexual partners. |
| Regular sexual partners | Individuals with whom participants maintained ongoing sexual relationships (≥3 months duration) characterized by mutual commitment, repeated engagement, and emotional connection |
| Casual sexual partners | Individuals engaged in transient sexual encounters without commitment or expectation of continuity (e.g., one-time partners, brief affiliations; typically, <3 months duration). |
| Gay social media/forums | Digital platforms (e.g., Blued) primarily used by MSM for: (1) partner-seeking, (2) community networking, (3) leisure activities. Quantified via self-reported usage frequency/purpose. |
| Gay bars/bathhouses | Physical venues catering predominantly to MSM, serving as social spaces facilitating partner-seeking opportunities. Assessed via attendance frequency and self-reported activities. |
| One-night stand | A single-episode sexual encounter initiated without prior relationship or intention of future contact. Defined behaviorally through participant self-report of unplanned encounters with strangers/casual acquaintances. |

Supplementary Table 2. Variable-Specific Missing Percentages [n(%)]

| Variable | Total | HIV-negative | HIV-positive |
| --- | --- | --- | --- |
|  | N=686 | N=348 | N=338 |
| Did you know the other person’s infection status before having sex? | 169 (24.6%) | 94 (27.0%) | 75 (22.2%) |
| Condom use in the past 6 months | 214 (31.2%) | 66 (19.0%) | 148 (43.8%) |
| Have your STDs been treated? | 607 (88.5%) | 316 (90.8%) | 291 (86.1%) |
| How many visits to gay bars in the past 6 months? | 405 (59.0%) | 214 (61.5%) | 191 (56.5%) |
| Have you had sex with someone you met at a gay bar? | 405 (59.0%) | 214 (61.5%) | 191 (56.5%) |
| How do you use condoms during sex in gay bars? | 664 (96.8%) | 336 (96.6%) | 328 (97.0%) |
| What’s the reason you don’t go to gay bars? | 281 (41.0%) | 134 (38.5%) | 147 (43.5%) |
| Do you have sex in a gay bathhouse? | 614 (89.5%) | 304 (87.4%) | 310 (91.7%) |
| Condom use during male sex in gay bathhouses | 671 (97.8%) | 342 (98.3%) | 329 (97.3%) |
| What are the reasons to not go to gay bathhouses? | 64 (9.3%) | 40 (11.5%) | 24 (7.1%) |
| How often have you visited gay social media or forums in the past 6 months? | 21 (3.1%) | 12 (3.4%) | 9 (2.7%) |
| What is your main purpose for visiting gay social software or forums? | 21 (3.1%) | 12 (3.4%) | 9 (2.7%) |
| Have you had sex with someone you met through an app or forum? | 83 (12.1%) | 41 (11.8%) | 42 (12.4%) |

* The above-mentioned variables contain a substantial proportion of missing values due to survey skip logic design, whereas no missing values exist in other non-listed variables.

Supplementary Table 3 Socio-demographic information by HIV status

|  |  | Total | HIV-negative | HIV-positive | *p*-value | d/V^*^ |
| --- | --- | --- | --- | --- | --- | --- |
|  |  | N=686 | N=348 | N=338 |  |  |
| Province (%) | | | | | |  |
|  | Shaanxi | 220(32.1) | 103(29.6) | 117(34.6) | 0.573 | 0.06 |
|  | Chongqing | 230(33.5) | 123(35.3) | 107(31.7) |  |  |
|  | Guangdong | 236(34.4) | 122(35.1) | 114(33.7) |  |  |
| Age (median [IQR]) | | 22.00[21.00, 24.00] | 22.00[20.00, 24.00] | 23.00[21.00, 25.00] | **0.007** | 0.14 |
| Level of study(%) | | | | | |  |
|  | Undergraduate | 591(86.2) | 294(84.5) | 297(87.9) | 0.430 | 0.05 |
|  | Postgraduate | 95(13.8) | 54(15.5) | 41(12.1) |  |  |
| Father’s education level(%) | | | | | |  |
|  | Middle school and below | 340(49.6) | 168(48.3) | 172(50.9) | 0.613 | 0.05 |
|  | High school | 207(30.2) | 102(29.3) | 105(31.1) |  |  |
|  | College degree and above | 139(20.3) | 78(22.4) | 61(18.0) |  |  |
| Mother’s education level(%) | | | | |  |  |
|  | Middle school and below | 403(58.7) | 207(59.5) | 196(58.0) | 0.730 | 0.04 |
|  | High school | 177(25.8) | 84(24.1) | 93(27.5) |  |  |
|  | College degree and above | 106(15.5) | 57(16.4) | 49(14.5) |  |  |
| Monthly expenditure (%) | | | | | |  |
|  | Below 1,000 RMB | 182(26.5) | 88(25.3) | 94(27.8) | 0.905 | 0.03 |
|  | 1000-2000 RMB | 363(52.9) | 187(53.7) | 176(52.1) |  |  |
|  | More than 2,000 RMB | 141(20.6) | 73(21.0) | 68(20.1) |  |  |
|  |  |  |  |  |  |  |
| Where did you live before attending college? | | | | | |  |
|  | Urban | 592(86.3) | 309(88.8) | 283(83.7) | 0.145 | 0.07 |
|  | Rural | 94(13.7) | 39(11.2) | 55(16.3) |  |  |

*d: Cliff's Delta effect size; V: Cramer's V coefficient.; For continuous variables, Mann-Whitney U tests were used for non-normally distributed and/or heteroscedastic data. For categorical variables, Barnard tests were employed for 2×2 contingency tables, and Fisher’s exact tests for larger contingency tables. Effect sizes were reported as Cliff's δ for continuous variables and Cramer's V for categorical variables. Multiple comparisons were corrected using the Benjamini & Hochberg method. Statistical significance was defined as p < 0.05 (two-tailed). The selected cutoff points were |Cliff's δ| ≥ 0.15 and Cramer's V ≥ 0.10, indicating a small effect size according to conventional guidelines.

Supplementary Table 4 HIV knowledge scores by HIV-status: correct responses

| Items | Total | HIV-negative | HIV-positive | *p*-value | V^*^ |
| --- | --- | --- | --- | --- | --- |
|  | N=686 | N=348 | N=338 |  |  |
| AIDS knowledge awareness rate(%):Correct responses ≥9 items | 517(75.4) | 256(73.6) | 261(77.2) | 0.476 | 0.04 |
| 1. Is AIDS an incurable and serious infectious disease? | 520(75.8) | 266(76.4) | 254(75.1) | 0.905 | 0.02 |
| 2. Is the HIV epidemic growing rapidly, in Chinese youth students through male-to-male sexual contact? | 490(71.4) | 244(70.1) | 246(72.8) | 0.730 | 0.03 |
| 3. Are MSM currently disproportionately affected by HIV in China? | 573(83.5) | 284(81.6) | 289(85.5) | 0.407 | 0.05 |
| 4. Can HIV infection status be determined by a person's appearance? | 613(89.4) | 304(87.4) | 309(91.4) | 0.183 | 0.07 |
| 5. Can daily life or academic contact transmit HIV? | 606(88.3) | 300(86.2) | 306(90.5) | 0.178 | 0.07 |
| 6. Does having other sexually transmitted infections increase HIV acquisition risk? | 406(59.2) | 206(59.2) | 200(59.2) | 1.000 | 0.00 |
| 7. Does consistent correct condom use reduce HIV transmission risk? | 656(95.6) | 331(95.1) | 325(96.2) | 0.730 | 0.03 |
| 8. Does using synthetic drugs (e.g., methamphetamine, ecstasy, ketamine) increase HIV risk? | 569(82.9) | 280(80.5) | 289(85.5) | 0.178 | 0.07 |
| 9. Should individuals seek HIV testing and counseling after high-risk behaviors (e.g., needle-sharing, unprotected sex)? | 653(95.2) | 327(94.0) | 326(96.4) | 0.267 | 0.06 |
| 10. Are rights to marriage, employment, and education legally protected for people living with HIV? | 510(74.3) | 257(73.9) | 253(74.9) | 0.905 | 0.01 |
| 11. Is intentional HIV transmission legally punishable? | 628(91.5) | 312(89.7) | 316(93.5) | 0.171 | 0.07 |

*V: Cramer's V coefficient.; For categorical variables, Barnard tests were employed for 2×2 contingency tables. Effect sizes were reported as Cramer's V for categorical variables. Multiple comparisons were corrected using the Benjamini & Hochberg method. Statistical significance was defined as p < 0.05 (two-tailed). The selected cutoff points were Cramer's V ≥ 0.10, indicating a small effect size according to conventional guidelines.

Supplementary Table 5 Main ways to meet sexual partners

|  |  | Overall | HIV-negative | HIV-positive | *p*-value | V^*^ |
| --- | --- | --- | --- | --- | --- | --- |
|  |  | N=686 | N=348 | N=338 |  |  |
| How do you mainly meet friends in the MSM community? (%) | | | | | |  |
|  | MSM on-campus | 237(34.5) | 153(44.0) | 84(24.9) | **<0.001** | 0.20 |
|  | MSM off-campus | 151(22.0) | 62(17.8) | 89(26.3) |  |  |
|  | Both are the same | 298(43.4) | 133(38.2) | 165(48.8) |  |  |
| The main way to meet male sexual partners^**^ | | | | | |  |
|  | Classmates | 149(21.7) | 87(25.0) | 62(18.3) | 0.107 | 0.08 |
|  | Internet | 647(94.3) | 324(93.1) | 323(95.6) | 0.403 | 0.05 |
|  | Friend introduction | 154(22.4) | 76(21.8) | 78(23.1) | 0.905 | 0.01 |
|  | Gay Bar | 23(3.4) | 10(2.9) | 13(3.8) | 0.730 | 0.03 |
|  | Other | 41(5.98) | 26(7.47) | 15(4.44) |  |  |
|  |  |  |  |  |  |  |
| Did you know the other person’s infection status before having sex?(%) | | | | | |  |
|  | Y | 88(17.0) | 64(25.2) | 24(9.1) | **<0.001** | 0.21 |
|  | N | 348(67.3) | 145(57.1) | 203(77.2) |  |  |
|  | Partially known | 81(15.7) | 45(17.7) | 36(13.7) |  |  |
|  |  |  |  |  |  |  |
| Do you want to know the other person’s infection status before having sex?(%) | | | | | |  |
|  | Y | 491(71.6) | 282(81.0) | 209(61.8) | **<0.001** | 0.21 |
| Do you protect yourself when having sex with a casual sex partner?(%) | | | | | |  |
|  | Y | 535(78.0) | 305(87.6) | 230(68.0) | **<0.001** | 0.24 |
|  | N | 33(4.8) | 12(3.4) | 21(6.2) |  |  |
|  | Sometimes | 118(17.2) | 31(8.9) | 87(25.7) |  |  |

*V: Cramer's V coefficient; For categorical variables, Barnard tests were employed for 2×2 contingency tables. Effect sizes were reported as Cramer's V for categorical variables. Multiple comparisons were corrected using the Benjamini & Hochberg method. Statistical significance was defined as p < 0.05 (two-tailed). The selected cutoff points were Cramer's V ≥ 0.10, indicating a small effect size according to conventional guidelines.

** Items marked with this superscript indicate questions where participants could select multiple responses. For these items, p-values are reported for each response category because each option was analyzed independently to assess significant differences between groups

Supplementary Table 6 Attitudes to high risk sex

|  |  | Overall | HIV-negative | HIV-positive | P-value | V^*^ |
| --- | --- | --- | --- | --- | --- | --- |
|  |  | N=686 | N=348 | N=338 |  |  |
| Attitude towards one-night stands(%) | | | | | |  |
|  | Approval | 132(19.2) | 84(24.1) | 48(14.2) | **0.019** | 0.13 |
|  | Neutrality | 353(51.5) | 165(47.4) | 188(55.6) |  |  |
|  | Be opposed to | 201(29.3) | 99(28.4) | 102(30.2) |  |  |
| Attitudes towards commercial sex(%) | | | | | |  |
|  | Approval | 51(7.4) | 34(9.8) | 17(5.0) | 0.078 | 0.10 |
|  | Neutrality | 300(43.7) | 157(45.1) | 143(42.3) |  |  |
|  | Be opposed to | 335(48.8) | 157(45.1) | 178(52.7) |  |  |
| Are you willing to be paid for sex?(%) | | | | | |  |
|  | Willing | 43(6.3) | 24(6.9) | 19(5.6) | 0.106 | 0.10 |
|  | Unwilling | 606(88.3) | 298(85.6) | 308(91.1) |  |  |
|  | It depends on the situation | 37(5.4) | 26(7.5) | 11(3.3) |  |  |
| Would you actively provide commercial sex in the future?(%) | | | | | |  |
|  | Y | 30(4.4) | 19(5.5) | 11(3.3) | 0.317 | 0.05 |
|  | N | 656(95.6) | 329(94.5) | 327(96.7) |  |  |

*V: Cramer's V coefficient; For categorical variables, Barnard tests were employed for 2×2 contingency tables. Effect sizes were reported as Cramer's V for categorical variables. Multiple comparisons were corrected using the Benjamini & Hochberg method. Statistical significance was defined as p < 0.05 (two-tailed). The selected cutoff points were Cramer's V ≥ 0.10, indicating a small effect size according to conventional guidelines.

Supplementary Table 7 Sexual behavior

|  |  | | | Overall | HIV-negative | | HIV-positive | | *p*-value | | d/V^*^ |
| --- | --- | --- | --- | --- | --- | --- | --- | --- | --- | --- | --- |
|  |  | | | N=686 | N=348 | | N=338 | |  | |  |
| Sex Role(%) | | | | | | | | | | |  |
|  | Insertive | | | 119(17.3) | 93(26.7) | | 26(7.7) | | **<0.001** | | 0.26 |
|  | Versatile | | | 362(52.8) | 173(49.7) | | 189(55.9) | |  | |  |
|  | Receptive | | | 205(29.9) | 82(23.6) | | 123(36.4) | |  | |  |
|  |  | | |  | |  | |  | |  |  |
| Age at first sex with men (median [IQR]) | | | | 18.00 [17.00, 19.00] | 18.00 [17.00, 20.00] | | 18.00 [17.00, 19.00] | | 0.202 | | -0.07 |
| First time men have sex with men(%) | | | | | | | | | | |  |
|  | Mutual consent | | | 605(88.2) | 317(91.1) | | 288(85.2) | | **0.045** | | 0.13 |
|  | Non-consensual | | | 22(3.2) | 9(2.6) | | 13(3.8) | |  | |  |
|  | Inducement by the other | | | 45(6.6) | 13(3.7) | | 32(9.5) | |  | |  |
|  | Others | | | 14(2.0) | 9(2.6) | | 5(1.5) | |  | |  |
| Whether condoms used when having sex with men for the first time(%) | | | | | | | | | | |  |
|  | Y | | | 454(66.2) | 236(67.8) | | 218(64.5) | | 0.407 | | 0.07 |
|  | N | | | 206(30.0) | 103(29.6) | | 103(30.5) | |  | |  |
|  | Don't remember | | | 26(3.8) | 9(2.6) | | 17(5.0) | |  | |  |
|  |  | | |  | |  | |  | |  |  |
| Total number of male sex partners (median [IQR]) | | | | 5 [3, 10] | 5 [3, 10] | | 5 [3, 10] | | 0.615 | | 0.04 |
|  | | | |  | |  | |  | |  |  |
| Number of male sex partners who are students (median [IQR]) | | | | 2 [1, 4] | 3 [1, 5] | | 2 [1, 4] | | **<0.001** | | -0.18 |
|  | | | |  | |  | |  | |  |  |
| Number of male sex partners who are regular sex partners(median [IQR]) | | | | 2 [1, 3] | 2 [1, 3] | | 2 [1, 3] | | 0.905 | | 0.01 |
|  | | | |  |  | |  | |  | |  |
| Number of male sexual partners in the past 6 months (%) | | | | | | | | | | |  |
|  | 0 | | | 232(33.8) | 74(21.3) | | 158(46.7) | | **<0.001** | | 0.28 |
|  | 1 | | | 273(39.8) | 157(45.1) | | 116(34.3) | |  | |  |
|  | 2 to 5 | | | 160(23.3) | 102(29.3) | | 58(17.2) | |  | |  |
|  | >5 | | | 21(3.1) | 15(4.3) | | 6(1.8) | |  | |  |
|  |  | | |  |  | |  | |  | |  |
| Number of male sexual partners in the past 6 months (median (Q1,Q3)) | | | | 1 [1, 1] | 1 [1, 1] | | 1 [0, 1] | | 0.905 | | 0.01 |
|  |  | | |  |  | |  | |  | |  |
| Have a regular sexual partner in the past 6 months (%) | | | | | | | | | | |  |
|  |  | | | 354(51.6) | 212(60.9) | | 142(42.0) | | **<0.001** | | 0.19 |
|  |  | | |  |  | |  | |  | |  |
| Having a casual sex partner in the past 6 months (%) | | | | | | | | | | |  |
|  |  | | | 199(29.0) | 129(37.1) | | 70(20.7) | | **<0.001** | | 0.18 |
| Condom use in the past 6 months (%) | | | | | | | | | | |  |
|  | Use it every time | | | 322(68.2) | 189(67.0) | | 133(70.0) | | 0.916 | | 0.04 |
|  | Used in most cases | | | 85(18.0) | 53(18.8) | | 32(16.8) | |  | |  |
|  | Use occasionally | | | 29(6.1) | 19(6.7) | | 10(5.3) | |  | |  |
|  | Never use | | | 36(7.6) | 21(7.4) | | 15(7.9) | |  | |  |
| Reasons for not using condoms during anal sex in the past 6 months^**^ | | | | | | | | | | |  |
|  | I think the other person is a healthy person | | | 70(10.2) | 47(13.5) | | 23(6.8) | | **0.019** | | 0.11 |
|  | I am in a fixed relationship with my partner | | | 63(9.2) | 40(11.5) | | 23(6.8) | | 0.106 | | 0.08 |
|  | There are no condoms around when having sex | | | 26(3.8) | 15(4.3) | | 11(3.3) | | 0.730 | | 0.03 |
|  | Condoms reduce pleasure | | | 22(3.2) | 16(4.6) | | 6(1.8) | | 0.109 | | 0.08 |
|  | The other party is unwilling to use them | | | 22(3.2) | 11(3.2) | | 11(3.3) | | 1.000 | | 0.00 |
|  | Others | | | 42(6.12) | 24(6.90) | | 18(5.33) | |  | |  |
| What is the longest period you have been with a male partner? (%) | | | | | | | | | | |  |
|  | 3 months | | | 118(17.2) | 70(20.1) | | 48(14.2) | | 0.145 | | 0.10 |
|  | 3 months-6 months | | | 89(13.0) | 45(12.9) | | 44(13.0) | |  | |  |
|  | 6 months-1 year | | | 142(20.7) | 78(22.4) | | 64(18.9) | |  | |  |
|  | More than 1 year | | | 337(49.1) | 155(44.5) | | 182(53.8) | |  | |  |
|  |  | | |  |  | |  | |  | |  |
| Do you want to have a long-term male sexual partner? (%) | | | | | | | | | | |  |
|  | Y | | | 599(87.3) | 315(90.5) | | 284(84.0) | | **0.047** | | 0.10 |
|  | N | | | 87(12.7) | 33(9.5) | | 54(16.0) | |  | |  |
| Sexual behavior experience (%)^**^ | | | | | | | | | | |  |
|  | Anal sex | | | 651(94.9) | 323(92.8) | | 328(97.0) | | **0.048** | | 0.10 |
|  | Oral sex | | | 606(88.3) | 317(91.1) | | 289(85.5) | | 0.078 | | 0.09 |
|  | Hand job | | | 348(50.7) | 198(56.9) | | 150(44.4) | | **0.006** | | 0.13 |
|  | Friction between thighs and penis | | | 167(24.3) | 100(28.7) | | 67(19.8) | | **0.033** | | 0.10 |
|  | Anal kissing | | | 106(15.5) | 63(18.1) | | 43(12.7) | | 0.145 | | 0.07 |
|  | Fingering | | | 69(10.1) | 35(10.1) | | 34(10.1) | | 1.000 | | 0.00 |
|  | Group sex | | | 28(4.1) | 15(4.3) | | 13(3.8) | | 0.905 | | 0.01 |
|  |  | | |  | |  | |  | |  |  |
| The main reasons for having a one-night stand (%)^**^ | | | |  |  | |  | |  | |  |
|  | To avoid disclosure of MSM identity | | | 21(3.1) | 3(0.9) | | 18(5.3) | | **0.004** | | 0.13 |
|  | Unable to find a male sex partner | | | 64(9.3) | | 29(8.3) | | 35(10.4) | 0.730 | | 0.03 |
|  | Different sexual partners bring novelty | | | 89(13.0) | 46(13.2) | | 43(12.7) | | 0.918 | | 0.01 |
|  | Regular male sexual partner, unable to be together often | | | 48(7.0) | 23(6.6) | | 25(7.4) | | 0.905 | | 0.02 |
|  | Physiological needs and sexual impulses | | | 353(51.5) | 177(50.9) | | 176(52.1) | | 0.905 | | 0.01 |
|  | Others | | | 60(8.7) | 37(10.6) | | 23(6.8) | |  | |  |
|  |  | | |  | |  | |  | |  |  |
| History of sexually transmitted infections (except HIV) (%) | | | | | | | | | | |  |
|  |  | | | 79(11.5) | 32(9.2) | | 47(13.9) | | 0.145 | | 0.07 |
|  |  | | |  |  | |  | |  | |  |
| Have your STDs been treated? (%) | | | | | | | | | | |  |
|  |  | | | 76(96.2) | 30(93.8) | | 46(97.9) | | 0.677 | | 0.11 |
|  |  | | |  |  | |  | |  | |  |
| Do you engage in any of the following specific sexual behaviors? (%)^**^ | | | | | | | | | | |  |
|  | | Sex under alcohol influence | 414(60.3) | | | 214(61.5) | | 200(59.2) | 0.730 | | 0.02 |
|  | | Sex under drug influence | 12(1.7) | | | 3(0.9) | | 9(2.7) | 0.171 | | 0.07 |
|  | | Group sex; | 62(9.0) | | | 34(9.8) | | 28(8.3) | 0.730 | | 0.03 |
|  | | Commercial sex | 15(2.2) | | | 7(2.0) | | 8(2.4) | 0.905 | | 0.01 |
|  | | Use of sex-enhancing substances | 299(43.6) | | | 144(41.4) | | 155(45.9) | 0.430 | | 0.05 |
|  | | Sexual intercourse with female partners | 80(11.7) | | | 40(11.5) | | 40(11.8) | 0.949 | | 0.01 |

*d: Cliff's Delta effect size; V: Cramer's V coefficient. For continuous variables, Mann-Whitney U tests were used for non-normally distributed and/or heteroscedastic data. For categorical variables, Barnard tests were employed for 2×2 contingency tables, and Fisher’s exact tests for larger contingency tables. Effect sizes were reported as Cliff's δ for continuous variables and Cramer's V for categorical variables. Multiple comparisons were corrected using the Benjamini & Hochberg method. Statistical significance was defined as p < 0.05 (two-tailed). The selected cutoff points were |Cliff's δ| ≥ 0.15 and Cramer's V ≥ 0.10, indicating a small effect size according to conventional guidelines.

** Items marked with this superscript indicate questions where participants could select multiple responses. For these items, p-values are reported for each response category because each option was analyzed independently to assess significant differences between groups

Supplementary Table 8 Social behavior related to sex for MSM: Gay bars and gay bathrooms

| value | level | Overall | HIV-negative | HIV-positive | *p*-value | d/V^*^ |
| --- | --- | --- | --- | --- | --- | --- |
|  |  | N=686 | N=348 | N=338 |  |  |
| Have you ever been to a gay bar?(%) | | | | | |  |
|  | Y | 281(41.0) | 134(38.5) | 147(43.5) | 0.407 | 0.05 |
|  | N | 405(59.0) | 214(61.5) | 191(56.5) |  |  |
| How many visits to gay bars in the past 6 months? (median [IQR]) | | 0.00 [0.00, 1.00] | 0.00 [0.00, 1.50] | 0.00 [0.00, 1.00] | **0.001** | -0.24 |
|  | |  |  |  |  |  |
| The main purpose of going to a gay bar (%)^**^ | | | | | |  |
|  | Find a sex partner | 9(1.3) | 6(1.7) | 3(0.9) | 0.730 | 0.04 |
|  | Leisure and entertainment | 203(29.6) | 94(27.0) | 109(32.2) | 0.269 | 0.06 |
|  | To meet new friends | 36(5.2) | 25(7.2) | 11(3.3) | 0.078 | 0.09 |
|  | To find a sense of belonging | 11(1.6) | 6(1.7) | 5(1.5) | 0.913 | 0.01 |
|  | Others | 68(9.9) | 35(10.1) | 33(9.8) |  |  |
|  |  |  |  |  |  |  |
| Have you had sex with someone you met at a gay bar?(%) | | | | | |  |
|  | Y | 22(7.8) | 12(9.0) | 10(6.8) | 0.730 | 0.04 |
|  |  |  |  |  |  |  |
| How do you use condoms during sex in gay bars?(%) | | | | | |  |
|  | Keep using | 16(72.7) | 9(75.0) | 7(70.0) | 0.905 | 0.18 |
|  | Used partially | 3(13.6) | 1(8.3) | 2(20.0) |  |  |
|  | Never use | 3(13.6) | 2(16.7) | 1(10.0) |  |  |
| What’s the reason you don’t go to gay bars?(%) | | | | | |  |
|  | Don't want to expose as gay | 21(5.2) | 11(5.1) | 10(5.2) | **0.004** | 0.20 |
|  | High cost | 11(2.7) | 5(2.3) | 6(3.1) |  |  |
|  | Unpleasant atmosphere | 221(54.6) | 98(45.8) | 123(64.4) |  |  |
|  | Others | 152(37.5) | 100(46.7) | 52(27.2) |  |  |
| Will you go to a gay bar in the future?(%) | | | | | |  |
|  | Y | 193(28.1) | 127(36.5) | 66(19.5) | **<0.001** | 0.22 |
|  | N | 312(45.5) | 125(35.9) | 187(55.3) |  |  |
|  | uncertain | 181(26.4) | 96(27.6) | 85(25.1) |  |  |
| Have you ever been to a gay bathhouse?(%) | | | | | |  |
|  | Y | 72(10.5) | 44(12.6) | 28(8.3) | 0.157 | 0.07 |
| Do you have sex in a gay bathhouses?(%) | | | | | |  |
|  | Y | 15(20.8) | 6(13.6) | 9(32.1) | 0.151 | 0.22 |
| Condom use during male sex in gay bathhouses (%) | | | | | |  |
|  | Keep using | 10(66.7) | 5(83.3) | 5(55.6) | 0.905 | 0.12 |
|  | Used partially | 3(20.0) | 0(0.0) | 3(33.3) |  |  |
|  | Never use | 2(13.3) | 1(16.7) | 1(11.1) |  |  |
| How many male sex partners have you met in a gay bathhouses ?(%) | | | | | |  |
|  | 0 | 673(98.1) | 343(98.6) | 330(97.6) | 0.730 | 0.05 |
|  | 1-5 | 8(1.2) | 4(1.1) | 4(1.2) |  |  |
|  | >5 | 5(0.7) | 1(0.3) | 4(1.2) |  |  |
| What are the reasons to not go to gay bathhouses? (%)^**^ | | |  |  |  |  |
|  | High risk of HIV/STI | 251 (40.4) | 127 (41.2) | 124 (39.5) | 0.904 | 0.02 |
|  | Poor sanitary conditions | 215(34.6) | 93(30.2) | 122(38.9) | 0.078 | 0.09 |
|  | Overly open behavior | 256(41.2) | 125(40.6) | 131(41.7) | 0.905 | 0.01 |
|  | Don’t want to reveal as gay | 46(7.4) | 16(5.2) | 30(9.6) | 0.112 | 0.08 |
|  | Worry about temptation | 32(5.1) | 11(3.6) | 21(6.7) | 0.179 | 0.07 |
|  | Others | 229(36.8) | 130(42.2) | 99(31.5) |  |  |

*d: Cliff's Delta effect size; V: Cramer's V coefficient. For continuous variables, Mann-Whitney U tests were used for non-normally distributed and/or heteroscedastic data. For categorical variables, Barnard tests were employed for 2×2 contingency tables, and Fisher’s exact tests for larger contingency tables. Effect sizes were reported as Cliff's δ for continuous variables and Cramer's V for categorical variables. Multiple comparisons were corrected using the Benjamini & Hochberg method. Statistical significance was defined as p < 0.05 (two-tailed). The selected cutoff points were |Cliff's δ| ≥ 0.15 and Cramer's V ≥ 0.10, indicating a small effect size according to conventional guidelines.

** Items marked with this superscript indicate questions where participants could select multiple responses. For these items, p-values are reported for each response category because each option was analyzed independently to assess significant differences between groups

Supplementary Table 9 Social behavior related to sex for MSM: gay social media

| value | level | Overall | HIV-negative | HIV-positive | *p*-value | d/V^*^ |
| --- | --- | --- | --- | --- | --- | --- |
|  |  | N=686 | N=348 | N=338 |  |  |
| Have you ever visited gay social media or forums?(%) | | | | | |  |
|  | Y | 665(96.9) | 336(96.6) | 329(97.3) | 0.780 | 0.02 |
|  |  |  |  |  |  |  |
| How often have you visited gay social media or forums in the past 6 months?(mean (SD)) | | 15.35(28.79) | 17.48(23.00) | 13.17(33.58) | **<0.001** | -0.22 |
|  | |  |  |  |  |  |
| Your main purpose for visiting gay social software or forums (%)^**^ | | | | |  |  |
|  | Find a sex partner | 243(36.5) | 137(40.8) | 106(32.2) | 0.078 | 0.09 |
|  | Leisure and entertainment | 360(54.1) | 188(56.0) | 172(52.3) | 0.730 | 0.04 |
|  | To meet new friends | 331(49.8) | 170(50.6) | 161(48.9) | 0.904 | 0.02 |
|  | To learn gay culture | 56(8.4) | 28(8.3) | 28(8.5) | 0.985 | 0.00 |
|  | Others | 85(12.8) | 48(14.3) | 37(11.2) |  |  |
| Have you had sex with someone you met through an app or forum?(%) | | | | | |  |
|  | Y | 603(90.7) | 307(91.4) | 296(90.0) | 0.730 | 0.02 |
| Condom use when having sex with men using software or forums(%) | | | | | |  |
|  | Keep using | 305(50.6) | 187(60.9) | 118(39.9) | **<0.001** | 0.21 |
|  | Used partially | 281(46.6) | 112(36.5) | 169(57.1) |  |  |
|  | Never use | 17(2.8) | 8(2.6) | 9(3.0) |  |  |
| Will you visit gay social software or forums in the future?(%) | | | | | |  |
|  | Y | 453(66.0) | 263(75.6) | 190(56.2) | **<0.001** | 0.21 |
|  | N | 98(14.3) | 30(8.6) | 68(20.1) |  |  |
|  | uncertain | 135(19.7) | 55(15.8) | 80(23.7) |  |  |

*d: Cliff's Delta effect size; V: Cramer's V coefficient. For continuous variables, Mann-Whitney U tests were used for non-normally distributed and/or heteroscedastic data. For categorical variables, Barnard tests were employed for 2×2 contingency tables, and Fisher’s exact tests for larger contingency tables. Effect sizes were reported as Cliff's δ for continuous variables and Cramer's V for categorical variables. Multiple comparisons were corrected using the Benjamini & Hochberg method. Statistical significance was defined as p < 0.05 (two-tailed). The selected cutoff points were |Cliff's δ| ≥ 0.15 and Cramer's V ≥ 0.10, indicating a small effect size according to conventional guidelines.

** Items marked with this superscript indicate questions where participants could select multiple responses. For these items, p-values are reported for each response category because each option was analyzed independently to assess significant differences between groups

Supplementary Table 10. Multivariable Logistic Regression Results for Association Between HIV Status and Binary Outcomes, Adjusting for Age. This table presents adjusted odds ratios (OR) and 95% confidence intervals (CI) from multivariable logistic regression analyses evaluating the independent association of HIV-positive status (vs. HIV-negative) with binary health outcomes after adjusting for age as a continuous covariate. All models included both predictors simultaneously (HIV status and age). Significant associations are denoted as follows: *P<0.05; **P<0.01; ***P<0.001.

| **Outcome** | **Predictor** | **β** | **SE** | **OR** | **OR (95% CI)** | ***p*-value** |
| --- | --- | --- | --- | --- | --- | --- |
| Do you want to know the other person’s infection status before having sex? (N vs Y) | HIV+ vs HIV- | 0.93 | -0.18 | 2.53 | [1.79, 3.60] | **<0.001^***^** |
|  | Age (per year) | 0.08 | -0.03 | 1.08 | [1.02, 1.14] | **0.004^**^** |
| Have a regular sexual partner in the past 6 months (N vs Y) | HIV+ vs HIV- | 0.78 | -0.16 | 2.19 | [1.61, 2.98] | **<0.001^***^** |
|  | Age (per year) | -0.02 | -0.03 | 0.98 | [0.93, 1.03] | 0.362 |
| Having a casual sex partner in the past 6 months (N vs Y) | HIV+ vs HIV- | 0.79 | -0.18 | 2.21 | [1.57, 3.12] | **<0.001^***^** |
|  | Age (per year) | 0.04 | -0.03 | 1.04 | [0.98, 1.10] | 0.220 |
| Reasons for not using condoms during anal sex in the past 6 months: I think the other person is a healthy person (Y vs N) | HIV+ vs HIV- | -18.05 | -2641.35 | 0.00 | - | 0.995 |
|  | Age (per year) | -0.27 | -0.26 | 0.76 | [0.41, 1.14] | 0.296 |
| Do you want to have a long-term male sexual partner? (N vs Y) | HIV+ vs HIV- | 0.59 | -0.24 | 1.80 | [1.14, 2.89] | **0.013*** |
|  | Age (per year) | 0.01 | -0.04 | 1.01 | [0.94, 1.08] | 0.762 |
| Sexual behavior experience: Anal sex (Y vs N) | HIV+ vs HIV- | -0.43 | -1.2 | 0.65 | [0.03, 5.30] | 0.720 |
|  | Age (per year) | -0.03 | -0.21 | 0.97 | [0.56, 1.34] | 0.877 |
| Sexual behavior experience: Hand job (Y vs N) | HIV+ vs HIV- | -17.53 | -3508.42 | 0.00 | - | 0.996 |
|  | Age (per year) | -0.06 | -0.35 | 0.94 | [0.32, 1.52] | 0.860 |
| Sexual behavior experience: Friction between thighs and penis (Y vs N) | HIV+ vs HIV- | -1.49 | -0.8 | 0.22 | [0.03, 0.91] | 0.061 |
|  | Age (per year) | 0.03 | -0.1 | 1.04 | [0.83, 1.23] | 0.724 |
| The main reasons for having a one-night stand: To avoid disclosure of MSM identity (Y vs N) | HIV+ vs HIV- | 1.18 | -1.16 | 3.24 | [0.41, 66.05] | 0.312 |
|  | Age (per year) | 0 | -0.16 | 1.00 | [0.68, 1.30] | 0.988 |

Supplementary Table 11. Multinomial Regression Results for Categorical Outcomes Comparing HIV-Positive Versus HIV-Negative Groups With Age Adjustment. Results of multinomial logistic regression analyses reporting relative risk ratios (RRR) and 95% confidence intervals (CI) for associations between HIV status and nominal health outcomes. All models included age as a continuous covariate to control for its confounding effect.

| **Outcome** | **Category** | **Predictor** | **β** | **SE** | **RRR** | **RRR (95% CI)** | ***p*-value** |
| --- | --- | --- | --- | --- | --- | --- | --- |
| How do you mainly meet friends in the MSM community? (vs MSM on-campus) | MSM off-campus | HIV+ vs HIV- | 0.90 | -0.22 | 2.46 | [1.61, 3.75] | **<0.001^***^** |
|  | Both are the same | HIV+ vs HIV- | 0.78 | -0.18 | 2.17 | [1.53, 3.09] | **<0.001^***^** |
|  | MSM off-campus | Age (per year) | 0.12 | -0.03 | 1.12 | [1.05, 1.20] | **0.001^***^** |
|  | Both are the same | Age (per year) | 0.08 | -0.03 | 1.08 | [1.02, 1.15] | 0.013^*^ |
| Did you know the other person’s infection status before having sex? (vs Y) | N | HIV+ vs HIV- | 1.28 | -0.26 | 3.59 | [2.14, 6.03] | **<0.001^***^** |
|  | Partially known | HIV+ vs HIV- | 0.74 | -0.33 | 2.10 | [1.10, 4.00] | 0.024^*^ |
|  | N | Age (per year) | 0.08 | -0.04 | 1.09 | [1.00, 1.18] | 0.050^*^ |
|  | Partially known | Age (per year) | 0.03 | -0.05 | 1.03 | [0.93, 1.15] | 0.535 |
| Do you protect yourself when having sex with a casual sex partner? (vs Y) | N | HIV+ vs HIV- | 0.84 | -0.37 | 2.31 | [1.11, 4.80] | **0.025**^*^ |
|  | Sometimes | HIV+ vs HIV- | 1.30 | -0.23 | 3.67 | [2.35, 5.74] | **<0.001^***^** |
|  | N | Age (per year) | 0.01 | -0.06 | 1.01 | [0.90, 1.13] | 0.877 |
|  | Sometimes | Age (per year) | 0.02 | -0.03 | 1.02 | [0.96, 1.09] | 0.538 |
| Attitude towards one-night stands (vs Approval) | Neutrality | HIV+ vs HIV- | 0.74 | -0.21 | 2.09 | [1.38, 3.18] | **0.001^***^** |
|  | Be opposed to | HIV+ vs HIV- | 0.65 | -0.23 | 1.92 | [1.22, 3.04] | **0.005**^**^ |
|  | Neutrality | Age (per year) | -0.06 | -0.03 | 0.94 | [0.88, 1.00] | 0.052 |
|  | Be opposed to | Age (per year) | -0.08 | -0.04 | 0.92 | [0.86, 0.99] | **0.020^*^** |
| Sex Role (vs Insertive) | Versatile | HIV+ vs HIV- | 1.39 | -0.25 | 4.02 | [2.47, 6.53] | **<0.001^***^** |
|  | Receptive | HIV+ vs HIV- | 1.74 | -0.27 | 5.71 | [3.38, 9.64] | **<0.001^***^** |
|  | Versatile | Age (per year) | -0.03 | -0.03 | 0.97 | [0.91, 1.03] | 0.334 |
|  | Receptive | Age (per year) | -0.08 | -0.04 | 0.93 | [0.86, 1.00] | **0.047^*^** |
| First time men have sex with men (vs Mutual consent) | Non-consensual | HIV+ vs HIV- | 0.52 | -0.44 | 1.69 | [0.71, 4.04] | 0.238 |
|  | Inducement by the other | HIV+ vs HIV- | 1.03 | -0.34 | 2.79 | [1.43, 5.44] | **0.003^**^** |
|  | Others | HIV+ vs HIV- | -0.55 | -0.57 | 0.58 | [0.19, 1.76] | 0.333 |
|  | Non-consensual | Age (per year) | -0.09 | -0.08 | 0.91 | [0.78, 1.07] | 0.270 |
|  | Inducement by the other | Age (per year) | -0.04 | -0.05 | 0.96 | [0.87, 1.07] | 0.442 |
|  | Others | Age (per year) | 0.07 | -0.08 | 1.08 | [0.93, 1.25] | 0.334 |
| Number of male sexual partners in the past 6 months (vs 0) | 1 | HIV+ vs HIV- | -1.06 | -0.19 | 0.35 | [0.24, 0.50] | **<0.001^***^** |
|  | 2 to 5 | HIV+ vs HIV- | -1.32 | -0.22 | 0.27 | [0.17, 0.41] | **<0.001^***^** |
|  | >5 | HIV+ vs HIV- | -1.69 | -0.51 | 0.18 | [0.07, 0.50] | **0.001^***^** |
|  | 1 | Age (per year) | 0.00 | -0.03 | 1.00 | [0.94, 1.06] | 0.922 |
|  | 2 to 5 | Age (per year) | 0.00 | -0.03 | 1.00 | [0.94, 1.07] | 0.986 |
|  | >5 | Age (per year) | 0.03 | -0.07 | 1.03 | [0.89, 1.18] | 0.704 |
| What’s the reason you don’t go to gay bars? (vs Don't want to expose as gay ) | High cost | HIV+ vs HIV- | 0.26 | -0.75 | 1.30 | [0.30, 5.66] | 0.724 |
|  | Unpleasant atmosphere | HIV+ vs HIV- | 0.33 | -0.46 | 1.39 | [0.57, 3.43] | 0.469 |
|  | Others | HIV+ vs HIV- | -0.54 | -0.47 | 0.58 | [0.23, 1.47] | 0.255 |
|  | High cost | Age (per year) | 0.03 | -0.13 | 1.03 | [0.80, 1.32] | 0.838 |
|  | Unpleasant atmosphere | Age (per year) | -0.02 | -0.08 | 0.98 | [0.83, 1.15] | 0.800 |
|  | Others | Age (per year) | -0.05 | -0.08 | 0.95 | [0.81, 1.13] | 0.584 |
| Will you go to a gay bar in the future? (vs Y) | N | HIV+ vs HIV- | 1.05 | -0.19 | 2.85 | [1.96, 4.15] | **<0.001^***^** |
|  | uncertain | HIV+ vs HIV- | 0.54 | -0.21 | 1.72 | [1.13, 2.62] | 0.011^*^ |
|  | N | Age (per year) | 0.02 | -0.03 | 1.02 | [0.96, 1.08] | 0.601 |
|  | uncertain | Age (per year) | -0.02 | -0.03 | 0.98 | [0.92, 1.05] | 0.615 |
| Condom use when having sex with men using software or forums (vs Keep using) | Used partially | HIV+ vs HIV- | 0.89 | -0.17 | 2.43 | [1.74, 3.40] | **<0.001^***^** |
|  | Never use | HIV+ vs HIV- | 0.59 | -0.50 | 1.81 | [0.68, 4.85] | 0.238 |
|  | Used partially | Age (per year) | -0.03 | -0.03 | 0.97 | [0.92, 1.03] | 0.336 |
|  | Never use | Age (per year) | -0.02 | -0.08 | 0.98 | [0.83, 1.15] | 0.775 |
| Will you visit gay social software or forums in the future? (vs Y) | N | HIV+ vs HIV- | 1.19 | -0.24 | 3.30 | [2.06, 5.30] | **<0.001^***^** |
|  | uncertain | HIV+ vs HIV- | 0.74 | -0.20 | 2.10 | [1.42, 3.12] | **<0.001^***^** |
|  | N | Age (per year) | -0.07 | -0.04 | 0.94 | [0.87, 1.01] | 0.092 |
|  | uncertain | Age (per year) | -0.06 | -0.03 | 0.95 | [0.89, 1.01] | 0.096 |

Supplementary Table 12. Multiple Linear Regression Coefficients for Continuous Outcomes Stratified by HIV Status with Age Covariate Adjustment. Estimated effects (β coefficients) with standard errors (SE) and 95% confidence intervals from multiple linear regression analyses modeling continuous outcomes. HIV status (positive/negative) and age (as continuous predictor) were incorporated simultaneously into all models.

| **Outcome** | **Predictor** | **β** | **SE** | **t-value** | **t-value (95% CI)** | ***p*-value** |
| --- | --- | --- | --- | --- | --- | --- |
| Number of male sex partners who are students | HIV+ vs HIV- | -2.12 | -0.54 | -3.93 | [-3.18, -1.06] | **<0.001^***^** |
|  | Age (per year) | 0.23 | -0.09 | 2.66 | [0.06, 0.40] | **0.008^**^** |
| Number of visits to gay bars in the past 6 months | HIV+ vs HIV- | -1.47 | -0.81 | -1.82 | [-3.07, 0.12] | 0.070 |
|  | Age (per year) | -0.14 | -0.12 | -1.17 | [-0.36, 0.09] | 0.241 |
| How often have you visited gay social media or forums in the past 6 months? | HIV+ vs HIV- | -4.40 | -2.24 | -1.96 | [-8.81, 0.00] | 0.050 |
|  | Age (per year) | 0.13 | -0.36 | 0.34 | [-0.59, 0.84] | 0.730 |
